# Differences in *Chlamydia trachomatis* seroprevalence between ethnic groups cannot be fully explained by socioeconomic status, sexual healthcare seeking behavior or sexual risk behavior: a cross-sectional analysis in the HEalthy LIfe in an Urban Setting (HELIUS) study

**DOI:** 10.1186/s12879-018-3533-7

**Published:** 2018-12-03

**Authors:** Sebastiaan H. Hulstein, Amy Matser, Catharina J. Alberts, Marieke B. Snijder, Martina Willhauck-Fleckenstein, Katrin Hufnagel, Maria Prins, Henry J. C. de Vries, Maarten F. Schim van der Loeff, Tim Waterboer

**Affiliations:** 1 Department of Infectious Diseases, Public Health Service of Amsterdam (GGD Amsterdam), Nieuwe Achtergracht 100, 1018 WT Amsterdam, The Netherlands; 2 Department of Internal Medicine, Amsterdam Institute for Infection and Immunity (AI&II), Academic Medical Center, Meibergdreef 9, 1105 AZ Amsterdam, The Netherlands; 3 Department of Public Health, Amsterdam Public Health Research Institute, Academic Medical Center, Meibergdreef 9, 1105 AZ Amsterdam, The Netherlands; 4 Department of Clinical Epidemiology, Biostatistics and Bioinformatics, Amsterdam Public Health Research Institute, Academic Medical Center, Meibergdreef 9, 1105 AZ Amsterdam, The Netherlands; 5 Molecular Diagnostics of Oncogenic Infections Division, German Cancer Research Center (Deutsches Krebsforschungszentrum DKFZ), Im Neuenheimer Feld 280, 69120 Heidelberg, Germany; 6 Department of Dermatology, Academic Medical Center, Meibergdreef 9, 1105 AZ Amsterdam, The Netherlands

**Keywords:** Ethnicity, *Chlamydia trachomatis*, Sexual healthcare seeking behavior, Socioeconomic status, Sexual risk behavior

## Abstract

**Background:**

In the Netherlands, there are strong disparities in *Chlamydia trachomatis* (CT) prevalence between ethnic groups. The current study aims to identify whether socioeconomic status, sexual risk behavior and sexual healthcare seeking behavior may explain differences in CT seroprevalence between ethnic groups.

**Methods:**

We used 2011–2014 baseline data of the HELIUS (HEalthy LIfe in an Urban Setting) study, a multi-ethnic population-based cohort study in Amsterdam, the Netherlands, including participants from Dutch, African Surinamese, South-Asian Surinamese, Ghanaian, Moroccan and Turkish origin. For this analysis, we selected sexually active, heterosexual participants aged 18–34 years old. CT seroprevalence was determined using a multiplex serology assay. The CT seroprevalence ratios between different ethnicities are calculated and adjusted for potential indicators of socioeconomic status, sexual risk behavior and sexual healthcare seeking behavior.

**Results:**

The study population consisted of 2001 individuals (52.8% female) with a median age of 28 years (IQR 24–31). CT seropositivity differed by ethnicities and ranged from 71.6% (African Surinamese), and 67.9% (Ghanaian) to 31.1% (Turkish). The CT seroprevalence ratio of African Surinamese was 1.72 (95% CI 1.43–2.06) and 1.52 (95% CI 1.16–1.99) of Ghanaian as compared to the Dutch reference group, after adjustment for socioeconomic status, sexual risk behavior and sexual healthcare seeking behavior.

**Conclusions:**

Indicators of socioeconomic status, sexual risk behavior, and sexual health seeking behavior could not explain the higher CT seroprevalence among African Surinamese and Ghanaian residents of Amsterdam.

**Electronic supplementary material:**

The online version of this article (10.1186/s12879-018-3533-7) contains supplementary material, which is available to authorized users.

## Short summary

A multi-ethnic cohort in Amsterdam, the Netherlands, showed that the disparate *Chlamydia trachomatis* seroprevalence between ethnic groups was not explained by socioeconomic status, sexual healthcare seeking behavior and sexual behavior.

## Background

*Chlamydia trachomatis* (CT) infections are a major public health concern. In many high income countries, including the Netherlands, CT prevalence, incidence and test positivity differs substantially across ethnic groups [1–5]. Urogenital CT infection is associated with sexual risk behavior (SRB) such as condomless sexual contact and higher number of sex partners [6, 7]. Some studies reported differences in these risk behaviors between ethnic groups [2, 8]. However, differences in individual risk behaviors fail to explain the differences in CT prevalence across ethnic groups [9–11]. Evidence from several population-based and STI clinic studies in the Netherlands suggested that sexual healthcare seeking behavior (sHSB) may play an important role in explaining the ethnic differences in CT infection rates [4]. Diagnosis and treatment of, especially asymptomatic, CT infections may be delayed by reduced sHSB, prolonging the time during which CT transmission is possible. This might lead to a higher CT prevalence in (sub)populations where members are characterized by low sHSB, high risk of CT infection, and have sex predominantly with members of the same group. Lower socioeconomic status (SES) is associated with lower sHSB [12]. In one study, CT infection was found to be associated with non-Dutch ethnicity and low SES [13]. In another study the uptake of CT screening tests was different between individuals with different ethnic backgrounds [4].

To better understand the contribution and interplay of SES, SRB and sHSB in their associations with CT and whether differences in these factors may explain differences in CT prevalence between ethnic groups, we performed a retrospective analysis of baseline data of a large multi-ethnic study: the HELIUS study. We examined whether SES, SRB and sHSB could explain the difference in heterosexual CT seroprevalence between ethnic groups in Amsterdam, the Netherlands.

## Material and methods

### Study population

The current study is based on baseline data from the HELIUS (HEalthy LIfe in an Urban Setting) study. The goals and design of the HELIUS study have been described before [14, 15]. In brief, HELIUS is a population-based study that includes individuals aged 18–70 years from the major ethnic groups living in Amsterdam (Surinamese, Turkish, Moroccan, Ghanaian and Dutch). Individuals were randomly selected, stratified by ethnicity, from the municipal registry of the city of Amsterdam and invited to participate in the study. The aim was to include similarly sized samples for each ethnic group, oversampling smaller groups to ensure this goal. Among each ethnic group, women were more likely to participate than men, and those who participated were slightly older than those who did not. Non-response analyses also indicated that, for each ethnic group, respondents and non-respondents did not differ regarding several SES indicators, suggesting representative samples for each ethnic group [15]. Baseline data collection took place from 2011 to 2015. Data were obtained by questionnaire and a physical examination, including collection of biological samples. Written informed consent was obtained from all participants. All HELIUS study protocols have been approved by the Ethics Review Board of the Academic Medical Center and are in accordance with the revised Declaration of Helsinki of 2000.

**Fig. 1:**
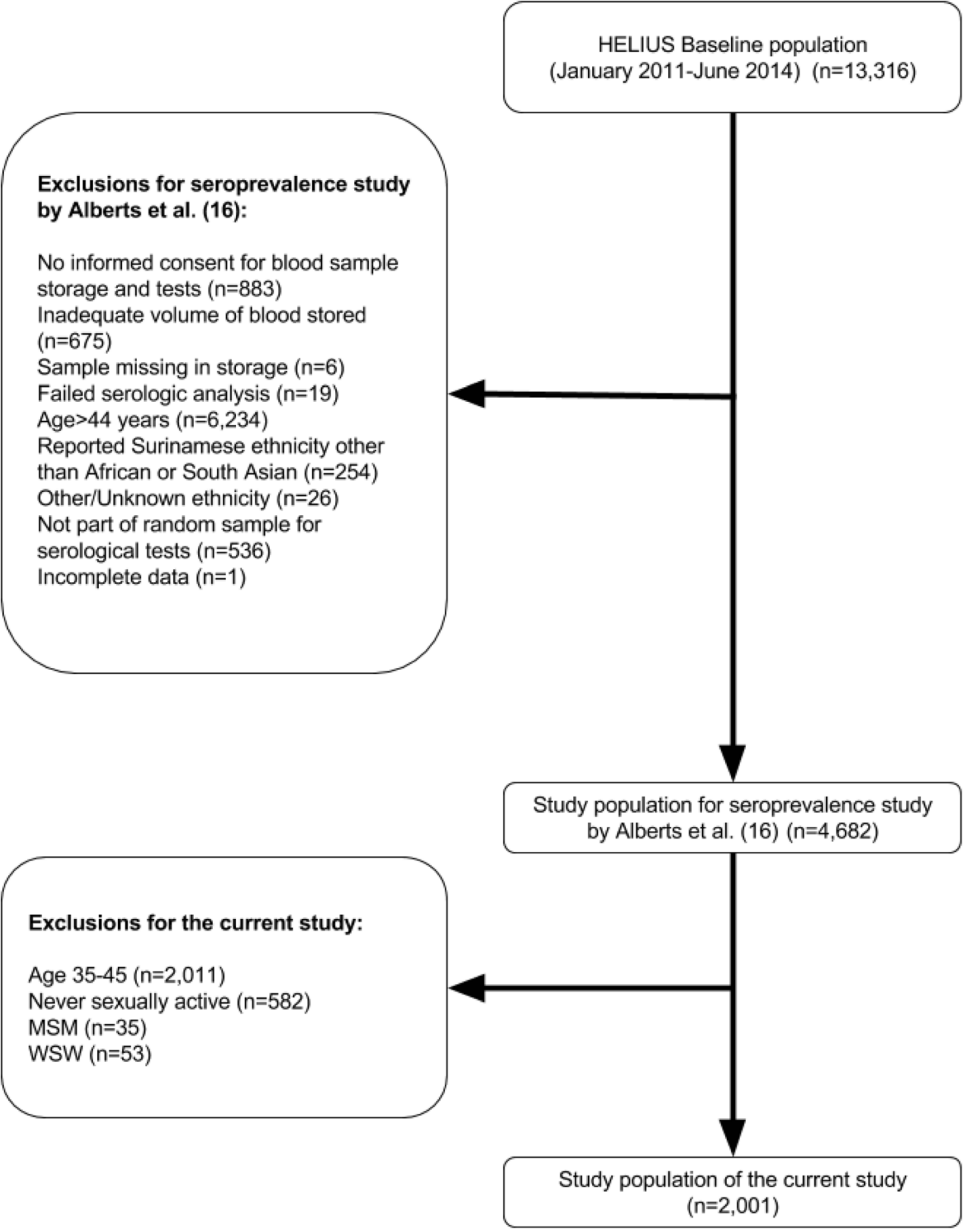
Flowchart of the selection process of participants for the current study

For the current study, a subset of the HELIUS baseline data and samples that were collected until June 2014 was used, on which CT serologic tests were performed. The selection procedure of the subsample has been described previously [16]. In short, among participants aged 18 to 44 years who gave informed consent for additional blood analyses and of whom an adequate volume of blood was available, a random sample per life year from each gender and ethnic group (Dutch, South-Asian Surinamese, African Surinamese, Ghanaian, Moroccan and Turkish ethnicity) was taken, resulting in a dataset of 4682 participants (Fig. 1) [16]. From this dataset we excluded men who have sex with men (MSM) and women who have sex with women (WSW) (based on self-reported behavior) and participants who had not yet had their sexual debut, because their Chlamydia infection risk differs from the general heterosexual population. All participants over 35 years old were excluded, as completion of questions on SRB was optional in this group and hence data were incomplete (Fig. 1).

### Ethnicity and migration status

Ethnicity was defined according to the country of birth of the participant as well as that of his/her parents [17]. Specifically, the HELIUS study invited participants of non-Dutch ethnic origin if the following criteria were met: 1) the participant and at least one parent were born abroad (first generation), or 2) the participant was born in the Netherlands, but both parents were born abroad (second generation). For participants of Surinamese origin, ethnicity was further classified according to self-reported ethnic origin (e.g. South-Asian, African, Javanese, or other). For the Dutch sample, people were invited who were born in the Netherlands and whose parents were born in the Netherlands.

### Chlamydia serological assay

To be able to take past CT infections into account, CT seroprevalence was determined as a proxy for lifetime CT exposure. Serum samples of fasting blood samples were tested for the presence of CT antibodies using a multiplex serology assay, developed at the German Cancer Research Center (DKFZ) in Heidelberg, Germany. This assay detects antibodies that bind to 7 CT antigens: the Major Outer Membrane Protein (MOMP) of serovars A, D, and L2, Translocated actin-recruiting phosphoprotein (Tarp, split into its N- and C-terminal fractions based on its size), and Porin B and Heat shock Protein-60 (Hsp60). The assay was validated against serum samples with known CT DNA status and a commercial ELISA (CT-ELISA, Medac, Wedel, Germany). The maximum of the 3 MOMP responses (MOMPmax) was used as a combined variable, and CT seropositivity was classified as an antibody response to 2 or more of the following antigens: Tarp-N, Tarp-C, PorB, and MOMPmax, or a median fluorescence intensity (MFI) > 1000 to MOMPmax alone, resulting in a sensitivity of 83% and a specificity of 87% for classification as CT seropositive (indicating CT exposure). Antibody cross-reactivity of *Chlamydia trachomatis* and *Chlamydophila pneumonia* was only minor, as indicated by correlation analyses (R^2^ < 0.05, data not shown) (Additional file 1: Appendix 1).

### SES, SRB, sHSB and other measurements

Information on demographics, SES, SRB, and sHSB were obtained by questionnaire. SES was determined by occupational level and educational level. Occupational level was classified according to the Standard Classification of Occupations of 2010 by Statistics Netherlands (CBS) [18]. This document provides an extensive systematic list of all professions in the Dutch system. Based on this document, occupational level was classified into (1) elementary, or lower, (2) intermediate and (3) higher or (4) academic, based on job title and job description, including a question on fulfilling an executive function (directing personnel). Educational level was based on the highest qualification attained, either in the Netherlands or in the country of origin, and it was categorized into three groups, namely (1) no or elementary schooling, or lower vocational or lower secondary schooling, (2) intermediate vocational, or intermediate or higher secondary schooling and (3) higher vocational schooling or university.

sHSB was based on self-reports of having been tested for HIV or other STIs in the preceding 6 months. SRB characteristics were the lifetime number of sex partners, age of sexual debut and self-reported condom use in the preceding 6 months with casual and steady sex partners, categorized as (1) no sexual contact in the past 6 months, (2) sex exclusively with a steady partner in the past 6 months (regardless of condom use), (3) consistent condom use with casual sex partners (regardless of sex with a steady partner), and (4) inconsistent or no condom use with casual sex partners (regardless of sex with a steady partner). Self-reported consistent condom use is defined as having always used a condom with a sexual partner. All degrees of self-reported inconsistency with condom use were defined as inconsistent condom use.

### Statistical analyses

The characteristics of the study population were described and differences between ethnic groups evaluated with χ^2^ tests for categorical variables or Kruskal-Wallis tests for continuous variables. The seroprevalence ratio (PR) of CT between the different ethnicities, and adjustments for potential explanatory indicators of SES, SRB, sHSB was calculated by Poisson regression analyses with robust variance [19].

For Poisson regression analyses, age was categorized into three strata: 18–24 years, 25–29 years and 30–34 years. The distribution of the number of lifetime sex partners was positively skewed and therefore transformed to its natural logarithm.

In univariable Poisson regression analyses with robust variance, we assessed the association of CT seropositivity with the variables selected in our theoretical model. These variables were age, sex and the indicators of SRB, SES and sHSB. Analyses were done overall, and stratified by ethnicity.

We assessed the CT seroprevalence ratios of the different ethnicities compared to the Dutch reference group, adjusting for potential explanatory indicators of SES, SRB, sHSB and the confounders age and gender in five steps. The first model included age and gender, the second age, gender and indicators of SRB, the third age, gender and indicators of SES, the fourth age, gender, and indicators of sHSB and the final model included age, gender and indicators of SRB, SES, and sHSB. In case of a PR > 1, a decrease of the CT seroprevalence ratio after adjustment with an indicator suggests an explanatory effect of that specific indicator for differences in CT seroprevalence between the compared groups. In case of a PR < 1, the same goes for an increase of CT seroprevalence ratio.

To assess the effect of migration generation on CT seroprevalence after adjustments for all indicators of SRB, SES and sHSB, we have performed a multivariable analysis stratified by ethnicity and migration generation.

Data were missing for some variables; as this was occurring in less than 5% of records per variable, we performed complete case analyses.

A statistical significance level of *P* < 0.05 was used. Statistical analyses were performed in STATA Intercooled 13.1 (College Station, Texas, USA).

## Results

### Study population selection and baseline characteristics including SES

Serological data were available for 4682 HELIUS participants. For the current analysis, 2011 participants aged 35 years or above, 582 participants who never had sexual intercourse, 35 MSM and 53 WSW were excluded. The resulting 2001 participants were included in the current study (Fig. 1).

In our study sample, the median age (28 years) of participants was similar across all ethnicities, and participants were mostly female (Table 1). Most participants of non-Dutch origin in the subsample were second generation migrants, except for Ghanaian participants who were mostly first generation migrants (78.2%). Overall, Dutch participants were higher educated and had higher occupational levels (Table 1).

**Table 1:** Characteristics of the study population (*n* = 2001)^a^, stratified by ethnicity

	Dutch (*n* = 392)		South Asian Surinamese (*n* = 373)		African Surinamese (*n* = 317)		Ghanaian (*n* = 193)		Turkish (*n* = 389)		Moroccan (*n* = 337)		Total (*n* = 2001)	
Demographics														
Sex [n,%] (*n* = 2001)														
Female	214	54.6%	185	49.6%	193	60.9%	121	62.7%	167	43.4%	174	51.6%	1056	52.8%
Migration generation [n,%] (*n* = 1609)														
First generation	N/A	N/A	82	22.0%	136	42.9%	151	78.2%	137	35.2%	117	34.7%	623	38.7%
Age in years [median, IQR] (*n* = 2001)	28	25–31	28	23–31	27	23–31	27	23–32	29	25–32	29	25–32	28	24–31
Socioeconomic status														
Educational level [n,%] (*n* = 1995)														
Low	21	5.4%	79	21.2%	51	16.1%	68	35.4%	143	36.9%	88	26.3%	450	22.6%
Intermediate	94	24.0%	163	43.5%	178	56.3%	89	46.4%	166	42.8%	157	46.8%	847	42.5%
High	277	70.7%	130	35.0%	87	27.5%	35	18.2%	79	20.4%	90	26.8%	698	35.0%
Occupational level [n,%] (*n* = 1717)														
Low	53	15.2%	125	38.1%	112	44.1%	94	64.0%	172	49.3%	112	38.6%	668	38.9%
Intermediate	64	18.3%	104	31.7%	90	35.4%	37	25.2%	110	31.5%	114	39.3%	519	30.2%
High	232	66.5%	99	30.2%	52	20.5%	16	10.9%	67	19.2%	64	22.1%	530	30.9%
Sexual risk behavior														
Lifetime number of sex partners [median, IQR] (*n* = 1901)	7	3–13	3	1–6	5	3–10	4	2–6	1	1–7	2	1–7	4	1–10
Age in years at sexual debut [median, IQR] (*n* = 1844)	17	16–19	17	16–19	16	15–18	17	16–19	18	16–22	18	16–22	17	16–19
Sexual contacts and condom use (preceding 6 months) [n,%] (*n* = 1836)														
No sexual contact	48	12.5%	67	18.8%	40	13.2%	43	26.5%	63	18.5%	61	19.7%	322	17.3%
Steady partner only^b^	239	62.1%	236	66.3%	185	61.1%	93	57.4%	215	63.1%	200	64.5%	1168	62.9%
Consistent condom use with casual partners^c^	30	7.8%	31	8.7%	37	12.2%	11	6.8%	32	9.4%	29	9.4%	170	9.2%
Inconsistent condom use with casual partners^c^	68	17.7%	22	6.2%	41	13.5%	15	9.3%	31	9.1%	20	6.5%	197	10.6%
Sexual health seeking behavior														
HIV testing (preceding 6 months) [n,%] (*n* = 1952)														
Yes	44	11.3%	37	10.0%	83	26.4%	41	21.8%	26	6.9%	29	8.7%	260	13.2%
STI testing (preceding 6 months) [n,%] (*n* = 1951)														
Yes	70	17.9%	49	13.2%	104	33.1%	40	21.2%	29	7.7%	45	13.5%	337	17.1%
Primary outcome														
*C. trachomatis* seropositivity [n,%] (*n* = 2001)														
Overall	147	37.5%	150	40.2%	227	71.6%	131	67.9%	121	31.1%	117	34.7%	893	44.6%
Female	79	36.9%	77	41.6%	147	76.2%	81	66.9%	46	27.2%	66	37.9%	496	47.0%
Male	68	38.2%	73	38.8%	80	64.5%	50	69.4%	75	34.1%	51	31.3%	397	42.0%

*IQR* Interquartile range, *N/A* Not available, *HIV* Human immunodeficiency virus, *STI* Sexually transmitted infection

Data are presented as n (%) or median (IQR)

^a^Numbers may not add up due to missing values

^b^Sex exclusively with one partner, regardless of condom use

^c^Has had a casual sex partner, irrespective of having had a steady partner

### Sexual risk behavior (SRB) and sexual healthcare seeking behavior (sHSB) between the different ethnicities

Several differences in SRB between the ethnic groups were observed. The median age at sexual debut was lowest among African Surinamese participants (16 years, IQR 15–18) and highest among Turkish and Moroccan participants (both 18 years, IQR 16–22).The median lifetime number of sex partners was highest among Dutch participants (7 sex partners, IQR 3–13), and lowest among Turkish participants (1 sex partner, IQR 1–7). Most individuals reported exclusive sexual contact with a steady partner (regardless of condom use). Compared to other participants, inconsistent condom use with casual sex partners was most frequent among Dutch and African Surinamese participants (Table 1).

sHSB was also dissimilar across the different ethnic groups. 13.2% of the study population reported having been tested for HIV and 17.1% for STI both in the preceding 6 months. The proportions that had been tested in the preceding 6 months for HIV (26.4%) and STI (33.1%) were highest among African Surinamese participants, and lowest among those of Turkish origin (Table 1).

#### Chlamydia seroprevalence

In the overall population, CT seroprevalence was 44.6%. CT seroprevalence was highest among African Surinamese (71.6%), followed by Ghanaian participants (67.9%). Overall CT seropositivity among male participants (42.0%) was slightly lower than among female participants (47.0%; *P* = 0.03).

### CT seroprevalence ratios without adjustments

In the total study population the risk of CT seropositivity was increased by being female, a higher lifetime number of sex partners, a lower age at sexual debut, and in the preceding 6 months: inconsistent condom use with casual sex partners, STI testing and HIV testing. Second generation migrants were less likely to having been exposed to CT than first generation migrants. A high level of education and a high level of occupation were significantly associated with not having been exposed to CT (Table 2).

**Table 2:** (Sero) prevalence ratios of CT for potential determinants, by ethnic group. Results of stratified unadjusted Poisson regression with robust variance

	Dutch (*n* = 392)	South Asian Surinamese (*n* = 373)	African Surinamese (*n* = 317)	Ghanaian (*n* = 193)	Turkish (*n* = 389)	Moroccan (*n* = 337)	Total (*n* = 2001)
	PR (95% CI)	PR (95% CI)	PR (95% CI)	PR (95% CI)	PR (95% CI)	PR (95% CI)	PR (95% CI)
Demographics							
Gender							
Male	1	1	1^*^	1	1	1	1^*^
Female	0.97 (0.75–1.25)	1.08 (0.84–1.37)	1.18 (1.01–1.38)	0.96 (0.79–1.18)	0.80 (0.59–1.09)	1.21 (0.90–1.63)	1.12 (1.01–1.23)
Migration generation							
First	N/A	1	1	1^*^	1	1	1^***^
Second	N/A	1.00 (0.74–1.35)	0.97 (0.85–1.12)	0.73 (0.53–0.99)	1.06 (0.77–1.45)	0.95 (0.70–1.29)	0.82 (0.74–0.91)
Age							
18–24 years	1	1	1	1	1	1	1
25–29 years	0.98 (0.70–1.36)	1.31 (0.93–1.83)	1.03 (0.87–1.22)	0.96 (0.73–1.27)	1.11 (0.73–1.68)	0.88 (0.58–1.32)	0.98 (0.86–1.11)
30–34 years	0.99 (0.70–1.38)	1.45 (1.05–2.00)^*^	1.03 (0.87–1.23)	1.17 (0.93–1.47)	1.08 (0.72–1.63)	0.96 (0.65–1.41)	1.01 (0.89–1.14)
Socioeconomic status							
Educational level							
Low	1	1	1	1	1	1	1
Intermediate	1.24 (0.65–2.39)	1.05 (0.76–1.44)	0.97 (0.81–1.17)	0.76 (0.62–0.94)^*^	0.83 (0.60–1.14)	1.01 (0.71–1.44)	0.99 (0.87–1.11)
High	1.09 (0.59–2.04)	0.93 (0.66–1.32)	0.91 (0.73–1.13)	0.79 (0.60–1.05)	0.66 (0.42–1.04)	0.95 (0.63–1.42)	0.84 (0.74–0.96)^*^
Occupational level							
Low	1	1	1	1	1	1	1
Intermediate	0.69 (0.43–1.10)	1.13 (0.83–1.53)	1.09 (0.92–1.28)	0.74 (0.53–1.02)	0.86 (0.61–1.21)	0.99 (0.68–1.36)	0.91 (0.80–1.03)
High	0.82 (0.58–1.15)	1.11 (0.81–1.52)	0.90 (0.71–1.14)	0.77 (0.49–1.20)	0.62 (0.38–1.01)	0.81 (0.52–1.27)	0.80 (0.70–0.91)^**^
Sexual risk behavior							
Number of lifetime sex partners^a^	1.32 (1.17–1.50)^***^	1.19 (1.06–1.33)^**^	1.05 (0.97–1.13)	1.02 (0.90–1.17)	1.12 (1.00–1.25)	1.12 (1.00–1.25)	1.16 (1.11–1.21)^***^
Age at sexual debut^b^	0.90 (0.84–0.98)^*^	0.97 (0.93–1.01)	0.99 (0.96–1.02)	0.96 (0.93–1.00)^*^	0.96 (0.92–1.00)^*^	0.96 (0.93–1.00)^*^	0.95 (0.93–0.96)^***^
Sexual partners and condom use (preceding 6 months)							
No sexual contact	1	1	1	1	1	1	1
Steady partner^c^	0.97 (0.64–1.48)	1.02 (0.72–1.43)	1.16 (0.89–1.52)	1.11 (0.86–1.43)	1.16 (0.75–1.79)	1.53 (0.95–2.45)	1.11 (0.96–1.29)
Consistent condom use^d^	1.32 (0.77–2.26)	1.16 (0.71–1.90)	1.40 (1.04–1.87)^*^	0.42 (0.16–1.13)	0.88 (0.43–1.79)	0.56 (0.20–1.54)	1.10 (0.88–1.36)
Inconsistent condom use ^d^	1.37 (0.87–2.16)	1.41 (0.86–2.29)	1.34 (1.00–1.80)	0.92 (0.58–1.47)	1.35 (0.75–2.45)	2.44 (1.38–4.30)^**^	1.42 (1.18–1.70)^***^
Sexual health seeking behavior							
HIV testing (preceding 6 months)							
No	1	1	1^*^	1	1^*^	1^*^	1^***^
Yes	1.24 (0.87–1.77)	1.38 (1.00–1.92)	1.16 (1.00–1.33)	1.12 (0.90–1.40)	1.68 (1.10–2.54)	1.56 (1.06–2.30)	1.51 (1.35–1.68)
STI testing (preceding 6 months)							
No	1^*^	1^***^	1	1	1	1	1^***^
Yes	1.38 (1.04–1.83)	1.64 (1.26–2.13)	1.07 (0.93–1.24)	1.15 (0.93–1.43)	1.48 (0.96–2.29)	1.18 (0.80–1.75)	1.45 (1.31–1.61)

*OR* Odds ratio, *CI* Confidence intervals, *N/A* not applicable, *HIV* Human immunodeficiency virus, *STI* Sexually transmitted infection, *CT Chlamydia trachomatis*

^a^Per increase of 1 natural log

^b^Per 1 year increase

^c^Sex with exclusive sex partner, regardless of condom use

^d^Has had a casual partner, irrespective of having had a steady partner

*overall *p* < 0.05

**overall *p* < 0.01

***overall *p* < 0.001

### Adjusted seroprevalence ratios of CT

The CT seroprevalence ratios (reference group: Dutch participants) were highest among African Surinamese (PR: 1.99; 95% CI 1.65–2.21) and Ghanaian participants (PR: 1.81; 95% CI 1.54–2.13) (Table 3). African Surinamese (adjusted PR (aPR): 1.72; 95% CI 1.43–2.06) and Ghanaians (aPR: 1.52; 95% CI 1.16–1.99) remained significantly more likely to be CT seropositive when adjusted for (1) age and gender, (2) age, gender and indicators of SRB, (3) age, gender and indicators of SES, (4) age, gender, and indicators of sHSB and (5) age, gender and indicators of SRB, SES, and sHSB .

**Table 3:** Adjusted (sero)prevalence ratios (PR) of CT by ethnicity, as compared to the Dutch reference group

Adjustments	South Asian Surinamese PR (95% CI)	African Surinamese PR (95% CI)	Ghanaian PR (95% CI)	Turkish PR (95% CI)	Moroccan PR (95% CI)
Unadjusted*	1.07 (0.90–1.28)	1.99 (1.65–2.21)	1.81 (1.54–2.13)	0.83 (0.68–1.01)	0.93 (0.76–1.12)
Model 1: Adjusted for age and gender	1.09 (0.91–1.30)	1.90 (1.64–2.20)	1.81 (1.54–2.13)	0.82 (0.67–1.00)	0.93 (0.76–1.13)
Model 2: Adjusted for age, gender and sexual risk behavior (SRB)^a^	1.29 (1.07–1.56)	1.87 (1.61–2.17)	1.83 (1.51–2.21)	1.01 (0.81–1.26)	1.13 (0.91–1.40)
Model 3: Adjusted for age, gender, and socioeconomic status (SES)^b^	1.11 (0.90–1.36)	1.76 (1.47–2.12)	1.58 (1.24–2.01)	0.75 (0.59–0.97)	0.89 (0.70–1.13)
Model 4: Adjusted for age, gender and sexual healthcare seeking behavior (sHSB)^c^	1.11 (0.93–1.33)	1.85 (1.59–2.14)	1.78 (1.51–2.10)	0.86 (0.70–1.05)	0.94 (0.77–1.14)
Model 5: Adjusted for age, gender, SRB, SES and sHSB^a, b, c^	1.27 (1.02–1.58)	1.72 (1.43–2.06)	1.52 (1.16–1.99)	0.87 (0.66–1.13)	1.09 (0.84–1.40)

*PR* (Sero)prevalence ratio, *CI* Confidence intervals

*Results from unadjusted Poisson regression with robust variance

^a^Sexual risk behavior includes sexual contacts and condom use in the preceding 6 months, the natural log of lifetime sex partners and age at sexual debut

^b^Socioeconomic status includes educational level and occupational level

^c^Sexual healthcare seeking behavior includes HIV testing and STI testing in the preceding 6 months

Being of Turkish and Moroccan origin did not significantly increase the risk of CT seropositivity in univariable analysis, and the additional, stepwise, adjustments had little effect on the risk. In univariable analysis, South Asian Surinamese were as likely to be CT seropositive as the Dutch. When adjusted for age, gender, SRB, SES and sHSB (Step 5), the CT seroprevalence was significantly higher than in the Dutch (Table 3).

The multivariable analysis stratified by migration generation showed that among South Asian Surinamese second generation migration status, among Ghanaians first, among African Surinamese first and second was statistically associated with CT seropositivity, as compared to the Dutch. Of note, among South Surinamese first generation migration status and among Ghanaian second was not significantly associated with CT seropositivity compared to the Dutch (Table 4).

**Table 4:** Adjusted CT seropositivity ratios of different ethnic groups (stratified by migration generation), as compared to Dutch. The analyses were adjusted for SRB, SES and sHSB

Ethnicity	PR + 95% CI
South Asian Surinamese- first generation	1.26 (0.89–1.79)
South Asian Surinamese- second generation	1.27 (1.02–1.59)*
African Surinamese- first generation	1.80 (1.47–2.20)*
African Surinamese- second generation	1.66 (1.36–2.02)*
Ghanaian- first generation	1.73 (1.31–2.29)*
Ghanaian- second generation	1.04 (0.67–1.63)
Turkish- first generation	0.91 (0.64–1.31)
Turkish- second generation	0.85 (0.64–1.13)
Moroccan- first generation	1.22 (0.86–1.71)
Moroccan- second generation	1.04 (0.79–1.38)

* Indicates a statistically significant result (*p* <0.05)

## Discussion

In this study we assessed the disparate CT seroprevalence and aimed to explain the observed differences among various ethnic groups in the Netherlands. The current study showed a higher seroprevalence of CT among participants of African Surinamese and Ghanaian ethnic origin compared to the Dutch population. The seroprevalence of CT among three ethnicities (South-Asian Surinamese, Turkish and Moroccan participants) did not differ from that in the Dutch. Our study showed that SRB, SES and sHSB could not explain the disparate CT seroprevalence between the African Surinamese and Ghanaian groups and the Dutch. Being first or second generation migrant may play a role in explaining the ethnic differences in CT seroprevalence, especially among Ghanaians.

This is the first study to investigate the role of sHSB on disparate CT seroprevalence between ethnic groups. Other studies observed similar differences in CT between ethnic groups [3, 4, 9, 13], but were not based on diagnosis of CT by serology. Like in other studies [9–11] SRB failed to explain the discrepancies, but unlike a study performed by Matser et al. [9], SES was not explanatory for differences in CT between ethnicities.

The strength of this study, based on data from the large scale, population-based HELIUS study, lies in the sample sizes of the major ethnic groups of interest and data availability on demographics, SES, sHSB and SRB. These factors enabled us to assess whether these variables may explain in part the association between ethnicity and CT infection.

Another strength of this study is that similar ethnic group sizes were ensured during inclusion of HELIUS and only very small differences exist between participants and non-participants. Thus, the study sample is well suited for comparison of seroprevalence between the different ethnic groups. As no attempt is made to estimate the overall CT seroprevalence of Amsterdam oversampling of groups is of no concern [15].

A major limitation is the use of CT serology as a surrogate of a recent CT infection. Firstly, the specificity to detect acute infection is not optimal (87%), as it detects exposure to a (past) CT infection, rather than a recent infection. Moreover, CT seropositivity can also be induced by other CT infections like trachoma, which is a relatively common eye disease in West Africa [20] and in rare cases by pneumonia and inclusion conjunctivitis [21]. It may be that first generation migrants of Western African origin (i.e. Ghanaian participants) have been exposed to eye infections caused by CT, inflating the CT seroprevalence of the Ghanaian study group. Cross reactivity with *C. pneumoniae,* a well known issue with ELISA based CT tests, was only minor (details in Additional file 1: Appendix 1).

A specificity of less than 100% means that some participants are wrongly regarded as CT-exposed. There is no reason to believe that this misclassification differs by ethnicity or sHSB. Such random misclassification of CT status dilutes the strength of the effect of a risk factor with the outcome, but does not lead to the false identification of risk factors [22]. The use of Pgp3 based CT serological assays may improve the inherent properties of a serological CT test, as it has a higher specificity for CT [23, 24]. Lastly, the sensitivity of the CT serology may be affected by waning antibodies to CT overtime. For the current serological test the degree of waning is unknown. However, very recently Horner et al. [23] have shown that the waning of CT Pgp3 antibodies is less than 5% over 12 years, which suggests only a very modest effect of waning of CT antibodies. Use of NAAT based diagnosis of CT, might have alleviated some of these limitations caused by the suboptimal sensitivity and specificity of serological tests, but such results were not available [25].

Another limitation of the study is that no assessment could be performed on other areas of health care seeking behavior than sHSB. This might introduce a selection bias as low sHSB can either be appropriate or inappropriate depending on CT risk, but the difference between the two types of low sHSB cannot be discerned independently without an indication of healthcare seeking behavior in general. Likewise, if information on healthcare seeking behavior in general was available, but on sHSB was lacking, a similar selection would occur. In the multivariable models, adjustment for SRB was performed which minimizes the risk of selection bias due to this mechanism in the current study.

The cross-sectional design of the current study limits the possibilities to establish the causal role of SES, SRB, and sHSB in the association between ethnicity and CT. Follow-up data of CT infections and risk could establish temporal, etiologic causations of ethnicity with CT.

Another potential limitation concerns the representativeness of the HELIUS cohort with regards to health seeking behavior. It might be that HELIUS study participants differ from the general population in terms of health awareness and consequently health seeking behavior. Unfortunately, no information on these variables was available from the HELIUS (non)-response analysis, so no reasonable conclusion can be made [15].

Studies performed in the United States suggest that differences in sexual mixing patterns (e.g. assortative and disassortative mixing) between African-American and the Caucasian population as well as sexual concurrency play major roles in establishing and maintaining STI rate disparities [26, 27]. It may be that factors associated with the sexual network structure, such as assortative mixing patterns and concurrency, contribute to the increased CT risk among individuals of African Surinamese and Ghanaian ethnic origin in the Netherlands [8, 9, 26–29]. HELIUS does not collect sexual network data, so we were unable to evaluate the role of (dis)assortative mixing or concurrency, which limits the study in its potential to examine possible explanations of disparities in CT prevalence between different ethnicities in the Netherlands.

A last potential limitation is the lack of data on vaginal microbiota composition in the current study. The composition of vaginal microbiota differs between ethnicities [30–32], and might explain differences in CT prevalence as vaginal dysbiosis increases susceptibility to STIs [33–35]. Dysbiosis may partially or completely be responsible for the differences between ethnicities.

## Conclusions

In conclusion, the current study shows that CT seroprevalence differs between ethnic groups in Amsterdam, the Netherlands. CT seropositivity is most common among people of African Surinamese and Ghanaian origin. The differences in CT seroprevalence could not be explained by differences in SRB, SES or sHSB between ethnic groups. To gain a better understanding of the factors that drive CT (sero)disparities in Amsterdam, additional research should be performed, directed at establishing accurate estimates of CT incidence and its explanatory factors, including sexual mixing networks and concurrency and possible biologic mechanisms.

## Additional file

Additional file 1: Appendix 1, **Figure S1.** Comparison of antibody detection in sera with defined cervical Ct-DNA status. **Figure S2.** Comparison of Ct multiplex serology and *C. trachomatis* p-Elisa (Medac) in 80 sera from Mongolian women. (DOCX 248 kb)

## Abbreviations

(a)PR: (adjusted) Seroprevalence ratio
CBS: Statistics Netherlands
CI: Confidence interval
CT: *Chlamydia trachomatis*
DKFZ: German Cancer Research Center
HELIUS: HEalthy Life In an Urban Setting
Hsp60: Heat shock protein 60
MOMP: Major outer membrane protein
MSM: Men who have sex with men
SES: Socioeconomic status
sHSB: Sexual health seeking behavior
SRB: Sexual risk behavior
Tarp: Translocated actin-recruiting phosphoprotein
WSW: Women who have sex with women
