# Supplementary material for: Differences in Chlamydia trachomatis seroprevalence between ethnic groups cannot be fully explained by socioeconomic status, sexual healthcare seeking behavior or sexual risk behavior: a cross-sectional analysis in the HEalthy LIfe in an Urban Setting (HELIUS) study
Source: BMC Infect Dis. 2018 Dec 3;18:612. doi: 10.1186/s12879-018-3533-7 (PMC6278015; doi:10.1186/s12879-018-3533-7)
Supplement: Supplementary file 1 — Appendix 1, Figure S1. Comparison of antibody detection in sera with defined cervical Ct-DNA status. Figure S2. Comparison of Ct multiplex serology and C. trachomatis p-Elisa (Medac) in 80 sera from Mongolian women. (DOCX 248 kb) [file 12879_2018_3533_MOESM1_ESM.docx]

**Understanding the differences in Chlamydia trachomatis seroprevalence between ethnic groups. A cross-sectional analysis in HELIUS**

Hulstein S.H., Matser A., Alberts C.J, Snijder M.B., Willhauck- Fleckenstein M., Hufnagel, K., Prins M., de Vries H.J.C., Schim van der Loeff M.F., Waterboer T.

**APPENDIX**

***C. trachomatis* Multiplex Serology (Ctms) validation**

With a newly developed *Chlamydia trachomatis* (Ct) multiplex serology assay (Ctms) we analyzed Ct antibody responses to seven previously described immunogenic Ct proteins: the major outer membrane proteins of serovars A, D, and L2 (MOMP-A, MOMP-D, MOMP-L2), Porin B (PorB), Heat shock protein 60 (Hsp60, variant 3) and the Translocated actin-recruiting phosphoprotein (Tarp)([1](#_ENREF_1)). To avoid insufficient expression of large fusion proteins, Tarp was expressed in N- and C-terminal fragments (Tarp-N, Tarp-C). Ctms was developed and performed as described by Waterboer et al. for Human Papillomavirus (HPV) serology.([2](#_ENREF_2)) Sera utilized for assay validation were part of a Mongolian population-based cross-sectional HPV prevalence study comprising 1002 women (median age 36 years, range 15 to 59)([3](#_ENREF_3)). The study was approved by the ethical review committees of the International Agency for Research on Cancer (IARC) and the Ministry of Health in Mongolia, and all study participants provided informed consent. In total, 985 of the 1002 women provided both serum (for antibody analysis) and cervical liquid-based cytology specimens (for DNA analysis) and were characterized for Ct-DNA positivity([4](#_ENREF_4)). Cut-off values for the individual antigens (MOMP-A, -D, -L2, Tarp-N and -C, PorB and Hsp60) were calculated from the median fluorescence intensity (MFI) values of sera from 85 Ct-DNA positive women (Ct-DNA+) and 29 Ct-DNA negative women (Ct-DNA-). The latter group was additionally restricted to young women (<22 years of age) having had at most one life-time sexual partner. Cut-off values were determined by Receiver Operating Characteristic (ROC) analysis maximizing sensitivity and specificity. Seropositivity for individual antigens was defined as antibody reactivity above the antigen-specific cut-off.

To monitor antibody cross-reactivity of *Chlamydia trachomatis* and *Chlamydophila pneumoniae*, one of the major problems addressed for Ct ELISA technology in the literature, we expressed the homologous genes from *Chlamydophila pneumoniae* as GST fusion proteins and assessed simultaneously the antibody responses for both genera. Correlation coefficient analyses (all R2 <0.05; data not shown) indicate only minor cross-reactivity for all four full length proteins used as antigens.

Sera from Ct-DNA+ women showed higher reactivity compared to those from Ct-DNA-negative women, with at least three-fold increased median MFI for all MOMP, Tarp and PorB proteins (all p<0.001) whereas Hsp60 antibody responses showed no significant difference between the groups (Fig. 1). Seroprevalence for single proteins was always higher in Ct-DNA+ women compared to Ct-DNA- women (all p<0.0001) except for Hsp60 (not significant). Of the seven antigens, all except Hsp60 were considered informative and thus included in Ctms. Based on antibody cross-reactivity, the highest MFI of the three MOMP antigens (A, D, L2) was reported as MOMPmax value in subsequent analyses.

Figure 1: Comparison of antibody detection in sera with defined cervical Ct-DNA status. Antibody reactivity to individual proteins (median fluorescence intensities, MFI) were measured in sera from women either Ct-DNA+ (n=85, Ref group +) or Ct-DNA- (n=29, <22 years, ≤ 1 lifetime sexual partner, Ref group -). Black lines indicate the antigen specific cut-offs determined by ROC analysis.

Comparison of antibody reactivity to multiple proteins showed that sera from Ct-DNA+ women reacted with a median of three Ct proteins, while sera from Ct-DNA- women reacted with a median of only one Ct protein. Among the 85 sera from Ct-DNA+ women, 28 (33%) recognized all four proteins, of which 90% (25/28) were highly reactive (>1000 MFI) with MOMPmax. Using Ct DNA status in the two reference groups as gold-standard, maximum values for sensitivity and specificity were achieved when Ct seropositivity was defined as antibody response to 2 or more individual proteins or to MOMPmax >1000 MFI alone (sensitivity 83% and specificity 87%, respectively).

A subset of 80 randomly selected sera of the Mongolian study was used for an additional validation study comparing the Ctms assay with the commercial peptide-based Medac Chlamydia trachomatis-IgG-pElisa (Ct-ELISA; Medac, Wedel, Germany), performed according to the manufacturer’s instructions. For 38 out of 80 women (48%), Ct-DNA status was known with 14 Ct-DNA- (18%) and 24 Ct-DNA+ (30%) women. A comparison of the two assays resulted in an overall agreement of 75% with 23 concordantly positives (28.8%), 37 concordantly negatives (46.2%) and 20 discordant (25%) observations. All discordant observations were Ct multiplex serology positive and Ct-ELISA negative (Fig. 2). Concordantly positive sera showed high reactivity in Ctms recognizing a median of 4 proteins. Among 37 concordantly negative sera, 28 (75%) did not recognize any protein in Ctms. Out of the 20 discordant Ct-ELISA negative but Ctms positive sera, 19 (95%) were reactive to ≥3 proteins (Fig. 2), and 12 of the discordant observations (60%) were Ct-DNA positive compared to 47.8% Ct-DNA+ in the concordantly positive group. Based on the high percentage of Ct-DNA+ women in conjunction with strong antibody responses in the discordant group, we conclude that these discordant sera were false-negative in the Ct-ELISA, thereby indicating a higher sensitivity of our newly developed Ctms assay in comparison to the Ct-ELISA.

**No of *C. trachomatis* proteins recognized**

**OD**

**ELISA**

**0**

**1**

**2***

**3**

**4**

**0.01**

**0.1**

**1**

**10**

+

-

+ 23 0

- 20 37

Ctms

ELISA

-

Ct ELISA -

Ct ELISA +

Ctms -

Ctms+

Figure 2: Comparison of Ct multiplex serology and *C. trachomatis* p-Elisa (Medac) in 80 sera from Mongolian women. Dashed lines indicate cut-offs for Ctms (vertical) and Ct-ELISA (horizontal).

The 2* category on the x-axis includes MOMP_max_ alone >1000 MFI. Ct-DNA status is indicated as black (Ct-DNA positive) and white circles (Ct-DNA negatives or untested). The inset shows the concordance of the Ct serostatus obtained by Ctms and Ct ELISA. All Ct-ELISA positives were positive with Ct multiplex serology. Of additional 20 Ctms positive but Ct-ELISA negative sera, 60% were Ct-DNA positive.

**References for supplementary text 1**

1. Hafner LM, Wilson DP, Timms P**.** Development status and future prospects for a vaccine against Chlamydia trachomatis infection. Vaccine. 2014;32(14):1563-71.

2. Waterboer T, Sehr P, Michael KM, et al. Multiplex human papillomavirus serology based on in situ-purified glutathione s-transferase fusion proteins. Clin Chem. 2005;51(10):1845-53.

3. Dondog B, Clifford GM, Vaccarella S, et al. Human papillomavirus infection in Ulaanbaatar, Mongolia: a population-based study. Cancer Epidemiol Biomarkers Prev. 2008;17(7):1731-8.

4. Schmitt M, Depuydt C, Stalpaert M, Pawlita M**.** Bead-based multiplex sexually transmitted infection profiling. J Infect. 2014;69(2):123-33.
